# Functional Characteristics of Aldehyde Dehydrogenase and Its Involvement in Aromatic Volatile Biosynthesis in Postharvest Banana Ripening

**DOI:** 10.3390/foods11030347

**Published:** 2022-01-26

**Authors:** Yoshinori Ueda, Wei Zhao, Hideshi Ihara, Yoshihiro Imahori, Eleni Tsantili, Sumithra K. Wendakoon, Alan Chambers, Jinhe Bai

**Affiliations:** 1 Center for Research and Development of Bioresources, Osaka Prefecture University, 1-1 Gakuen-cho, Nakaku, Sakai 599-8531, Osaka, Japan; 2 U.S. Horticultural Research Laboratory, Agricultural Research Service, U.S. Department of Agriculture, 2001 S. Rock Rd., Ft Pierce, FL 34945, USA; Wei.zhao@usda.gov; 3 Department of Biological Science, Graduate School of Science, Osaka Prefecture University, 1-1 Gakuen-cho, Nakaku, Sakai 599-8531, Osaka, Japan; ihara@b.s.osakafu-u.ac.jp; 4 Graduate School of Life and Environmental Science, Osaka Prefecture University, 1-1 Gakuen-cho, Nakaku, Sakai 599-8531, Osaka, Japan; imahori@plant.osakafu-u.ac.jp; 5 Laboratory of Pomology, Department of Crop Science, Agricultural University of Athens, Iera Odos 75, 118 55 Athens, Greece; etsantili@aua.gr; 6 Department of Bioresource Science, Faculty of Agriculture, Ryukoku University, 1-5 Yokotani, Seta Oe-cho, Otsu 520-2194, Shiga, Japan; swendakoon@agr.ryukoku.ac.jp; 7 Tropical Research and Education Center, University of Florida, 18905 SW 280th St., Homestead, FL 33031, USA; ac@ufl.edu

**Keywords:** *Musa* AAA, ALDH, aroma volatile, ester, enzyme characteristics

## Abstract

Butanol vapor feeding to ripe banana pulp slices produced abundant butyl butanoate, indicating that a portion of butanol molecules was converted to butanoate/butanoyl-CoA via butanal, and further biosynthesized to ester. A similar phenomenon was observed when feeding propanol and pentanol, but was less pronounced when feeding hexanol, 2-methylpropanol and 3-methylbutanol. Enzymes which catalyze the cascade reactions, such as alcohol dehydrogenase (ADH), acetyl-CoA synthetase, and alcohol acetyl transferase, have been well documented. Aldehyde dehydrogenase (ALDH), which is presumed to play a key role in the pathway to convert aldehydes to carboxylic acids, has not been reported yet. The conversion is an oxygen-independent metabolic pathway and is enzyme-catalyzed with nicotinamide adenine dinucleotide (NAD^+^) as the cofactor. Crude ALDH was extracted from ripe banana pulps, and the interference from ADH was removed by two procedures: (1) washing off elutable proteins which contain 95% of ADH, but only about 40% of ALDH activity, with the remaining ALDH extracted from the pellet residues at the crude ALDH extraction stage; (2) adding an ADH inhibitor in the reaction mixture. The optimum pH of the ALDH was 8.8, and optimum phosphate buffer concentration was higher than 100 mM. High affinity of the enzyme was a straight chain of lower aldehydes except ethanal, while poor affinity was branched chain aldehydes.

## 1. Introduction

Aldehyde dehydrogenases (ALDH, aldehyde:NAD(P)^+^ oxidoreductases, EC 1.2.1) use nicotinamide adenine dinucleotide (NAD^+^) or nicotinamide adenine dinucleotide phosphate (NADP^+^) as a cofactor to convert aldehydes to their corresponding carboxylic acids plus NADH or NADPH. ALDH in plants are currently receiving considerable attention because they are involved in processing many aldehydes that serve as biogenic intermediates in a wide range of metabolic pathways [1]. They often function as an ‘aldehyde scavenger’, thus removing reactive aldehydes generated during the oxidative degradation, especially under environmental stress, such as exposure to salinity, drought, cold, and heat [1].

The highly abundant volatiles in fresh bananas are aldehydes, ketones, alcohols, carboxylic acids, and esters. It has been recognized that the production of straight chain alcohols, aldehydes, ketones, and acids in fruit is largely derived from α-oxidation, β-oxidation, or the lipoxygenase pathway [2,3]. Branched chain volatiles are derived from branched chain amino acids [4,5]. Recently, Sugimoto et al. [6,7] proposed that some branched and straight chain alcohols and acids come from the citramalate pathway.

**Figure 1:**
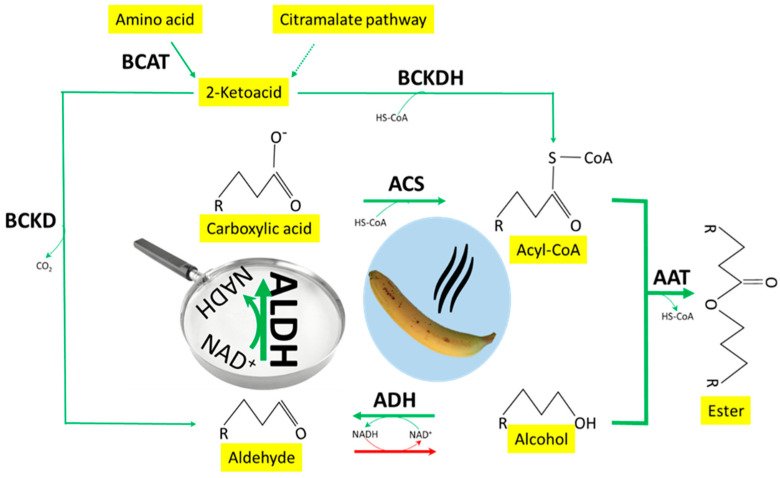
Role of aldehyde dehydrogenase (ALDH) in the dehydrogenation from aldehydes to carboxylic acids and the entire ester production pathways. ADH, alcohol dehydrogenase; ACS, acyl-CoA synthetase; AAT, alcohol acyl-CoA transferase; BCAT, branched-chain amino transferase; BCKD, branched-chain α-ketoacid decarboxylase; BCKDH, branched-chain α-ketoacid dehydrogenase.

Many enzymes regarding the conversions between volatile alcohols, aldehydes/ketones, acids, and esters have been studied extensively in fruits and plants, and/or adapted from microorganism studies [8,9,10]. In banana fruit ripening, gene expression and enzyme activity of alcohol dehydrogenase (ADH, short and medium chains), which facilitates the interconversion between alcohols to aldehydes [11,12], acyl-CoA synthetase (ACS), which activates carboxylic acids to acyl-CoAs [13] thus can be used to biosynthesize esters, and alcohol acetyl transferase (AAT), which catalyzes ester biosynthesis, have been intensively studied (Figure 1) [14,15,16,17]. ALDH dehydrogenizes aldehydes to carboxylic acids in different plant tissues [1,18]. However, to our knowledge, there is no report on the role of ALDH in volatile flavor metabolism in fruits.

Beekwilder, Alvarez-Huerta et al. [16] showed that when incubating petunia leaves with 3-methylbutanol vapor for 24 h, 3-methylbutyl 3-methylbutanoate was the dominant volatile, in addition to 3-methylbutanol in the headspace, indicating a strong dehydrogenization of 3-methylbutanol to 3-methylbutanal, further to 3-methylbutanoic acid, then passing through 3-methylbutanoyl-CoA, and finally synthesizing 3-methylbutyl 3-methylbutanoate, catalyzed by ADH, ALDH, ACS, and AAT, respectively [13,14,15,16].

The purpose of the current study was to determine the enzyme which converts volatile straight and branched aldehydes to carboxylic acids, to identify their involvement in volatile metabolisms in banana fruit (Figure 1), and to reveal the functional characteristics of ALDH. Due to both ADH and ALDH activities being determined by the change in NADH concentration, efforts were made to minimize the effect of ADH [19].

## 2. Materials and Methods

### 2.1. Chemicals and Reagents

Folin and Ciocalteu’s phenol reagent and 2-keto-4-methylpentanoic acid were obtained from Sigma-Aldrich (Tokyo, Japan). Polyclar-VT was purchased from Gokyo (Osaka, Japan). Bovine serum albumin, dithiothreitol (DTT), nicotinamide adenine dinucleotide (NAD^+^), nicotinamide adenine dinucleotide phosphate (NADP^+^), and the reduced forms NADH and NADPH, tris(hydroxymethyl)aminomethane (Tris), 2-[4-(2,4,4-trimethylpentan-2-yl)phenoxy]ethanol (Triton X100), 4-methyl pyrazole, sodium hydroxide (NaOH), phosphoric acid, C2–C6 branched and straight chain alcohols, aldehydes, and carboxylic acids were purchased from Wako (Osaka, Japan).

### 2.2. Plant Materials

Banana fingers (*Musa* spp. AAA group, Cavendish subgroup) ripened to “yellow with green tips”, “yellow”, or “yellow, flecked with brown”, unless otherwise stated [20], were produced in the Philippines and purchased from local grocery stores, and experiments were conducted in the Osaka Prefecture University lab. For ripening stage influence experiments, unripe fruit were purchased and ripening was triggered by 100 µL L^−1^ of ethylene at 20 °C for 24 h. For all samples, each replicate contained five fingers, and each treatment had three replicates. For fruit pulp slice samples, cylindrical pulp discs were taken from the central section of a banana finger and further divided into 4 wedges. Pulps were homogenized in some experiments.

### 2.3. Substrate Feeding Experiments

#### 2.3.1. Feeding of Alcohols and 2-Keto-4-Methylpentanoic Acid

**Figure 2:**
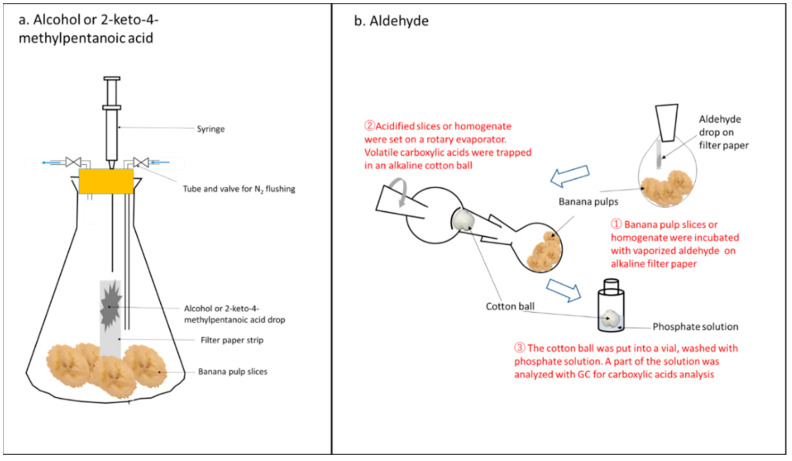
Feeding experiments procedures. (**a**) Feeding alcohol or 2-keto-4-methylpentanoic acid feeding to banana pulps to produce alcohol/aldehyde/esters in air or nitrogen gas. (**b**) Incubating aldehyde vapor with banana pulps to produce corresponding carboxylic acid, and separation of the carboxylic acid from pulps.

The feeding experiments were conducted by incubating 10 g of banana pulp slices with different exogenous substrates: 50 µmol of C3–C6 straight chain or branched alcohols, or 2-keto-4-methylpentanoic acid, in a 150-mL conical flask for 90 min at 35 °C. The substrates were spotted onto a 40 × 5 mm filter paper strip prior to incubation to enhance evaporation (Figure 2a). After incubation, 1 mL of headspace gas was taken with a glass syringe, and analyzed by gas chromatography (GC, Shimadzu GC-15A, Kyoto, Japan) to target alcohols, aldehydes, or esters. A flame ionization detector was equipped with a packed glass column (2 m × 3 mm) with 20% of tween 20 or 5% of polyethylene glycol 6000. Injector and detector temperatures were 190 °C, and oven temperature was 60–120 °C, depending on the molecular weight of the target esters. Flow rate of carrier nitrogen gas was 20 mL min^−1^ and flaming hydrogen gas was 30 mL min^−1^.

#### 2.3.2. Feeding of Aldehydes and Trapping of Carboxylic Acids

When feeding experiments were conducted by incubating 10 g of banana pulp slices with 50 µmol of exogenous C2–C6 straight chain or branched aldehydes, the incubation conditions were similar to Section 2.3.1 (Figure 2a,b). Due to the targeted products being carboxylic acids, specific procedures were applied as Figure 2b. Filter paper strips treated with NaOH (1 M) were dried before spotting exogenous substrate aldehydes to remove any possible acid impurities in the substrate aldehydes. A recovery flask (250-mL) was used for the incubation. After incubation, the pulp slices were well soaked with 1.5 mL of phosphoric acid (0.4 M) in the flask. Then, the flask was attached to a rotary evaporator in which a cotton ball was inserted at back-flow prevention glass wear. The cotton ball for the acid trap was previously treated with NaOH (0.1 M) and dried. After rotary evaporation (40 °C for 60 min), the cotton ball was put into a 17 mL vial and then mixed well with 5 mL of phosphoric acid (0.4 M). For ethanoic acid measurement, 5 mL of acidified ether was used instead of the phosphoric acid solution. For the phosphoric acid solution with carboxylic acids, 1 µL was injected into GC to determine the targeted carboxylic acids (Figure 2b). The GC conditions were: a glass column (3 m × 3 mm) of SP1200 (10%) + phosphoric acid (1%), and oven temperature 60–120 °C depending on molecular weight of the target acid. The recovery rate of this method was 53.3% ± 3.2 for ethanoic acid, 81.4% ± 4.7 for propanoic acid, 92.6 ± 1.6 for 2-methylpropanoic acid, 95.6 ± 6.1 for butanoic acid, 97.2% ± 7.4 for 3-methylbutanoic acid, 94.9% ± 7.2 for pentanoic acid, and 97.6% ± 1.3 for hexanoic acid (n = 3).

The same method as mentioned above was used for the feeding of aldehydes and trapping of acids when pulp homogenate was used instead of pulp slices (Figure 2b). Ten grams of pulp slices were macerated together with 10 mL of phosphate buffer (0.2 M, pH 8.8), 0.8 g of Polyclar-VT, 1.0 mg of DTT, and 3 g of quartz sand with a mortar and pestle in an ice bath. The pH of the homogenate was adjusted to pH 8.8 with 0.1 M NaOH just after macerating.

#### 2.3.3. Setting and Monitoring of O_2_ and CO_2_ in Incubation

To determine the effect of oxygen on the volatile metabolism, anaerobic conditions were created by flushing the conical flasks with pure nitrogen gas (Figure 2a). The headspace gas sample, 1 mL, was tested to confirm the conditions by using GC-thermal conductivity detector (TCD). A stainless column packed with active charcoal (4 m × 3 mm) was used for oxygen and a stainless column with active aluminum (1 m × 1 mm) was used for carbon dioxide analysis. The flow rate of carrier nitrogen was 10 mL min^−1^, and oven temperature was 50 °C.

Headspace gas samples during incubation were tested periodically to confirm that there were no severe anaerobic conditions, O_2_ > 15 kPa and CO_2_ < 5 kPa when using air as the incubation gas.

### 2.4. Crude ALDH Extraction from Banana Pulps, and Functional Characteristics of the Enzyme

#### 2.4.1. Crude Protein Extraction

Ten grams of banana pulps were macerated together with 10 mL of Tris buffer (0.2 M, pH 8.8), 0.8 g of Polyclar-VT, 1.0 mg of DTT, and 3 g of quartz sand with a mortar and pestle in an ice bath for 5 min. The final homogenates were adjusted to pH 8.5. From this point, the procedures were conducted under 4 °C in an ice bath. The homogenates were centrifuged at 8300× *g* for 15 min, and supernatants were collected. The pellets were carefully resuspended into 30 mL of Tris buffer (0.1 M, pH 8.8), and centrifuged again in the same condition. The first and second supernatants were merged for ALDH activity test (protein-S). The pellets were resuspended into 30 mL of Tris buffer (0.1 M, pH 8.8, containing 1.0 mg of DTT) with the assistance of ultrasonic wave for 10 min. Then, centrifugation (8300× *g* for 15 min) was applied. The supernatants were used for pellet ALDH activity test (protein-P).

For each of the above supernatants (protein-S and protein-P), solid ammonium sulfate was added to the supernatants (80% saturation: 5.61 g 10 mL^−1^). After stirring for 2 h, the precipitations were separated by centrifugation at 25,000× *g* for 15 min. The precipitations were solubilized by 10 mL of Tris buffer (0.01 M, pH 8.8) with DTT (1 mg) and dialyzed by using a total of 2 L of Tris buffer (0.01 M, pH 8.8) for 3 h where the buffer was renewed twice. After dialysis, the insoluble pellets were removed by centrifugation at 25,000× *g* for 15 min, and the supernatants were collected as crude protein-S and protein-P, respectively.

A crude protein-PT sample was also prepared to test whether Triton X-100 nonionic surfactant improves protein extraction and enzyme activity of crude protein-P. The procedures were similar to the above for protein-P, and the only change was to add 0.1% Triton X-100 to the Tris buffer during the ultrasonic treatment.

The crude proteins were stored at −20 °C until enzyme characteristics analysis. The activity was stable for at least one month under DTT condition.

#### 2.4.2. Functional Characteristics of ALDH

Substrate specificity: reaction mixture (2 mL) included 1660 µL of phosphate buffer (0.2 M, pH 8.8), 100 µL of low C2–C4 aldehydes (50 mM), 100 µL of crude protein extraction (protein-S, -P or -PT), 40 µL of NAD^+^ (50 mM), with or without 100 µL of 4-methyl pyrazole (100 mM). For C5–C6 aldehydes, 500 µL (10 mM) was added with reduced volume of buffer (1260 µL). ALDH activity was determined by monitoring the increase in NADH at 340 nm at 35 °C by a spectrophotometer with a 10 mm path length UV cuvette. Km value of NAD^+^ was measured with butanal as the substrate. An extinction coefficient of 6.2 mM^−1^ cm^−1^ was used for the activity calculation.

Optimum pH and buffer concentration: ALDH activity was determined by monitoring the increase in NADH at 340 nm at 35 °C by a spectrophotometer, as described above. Butanal was used as the substrate. For the optimum pH searching, bicine buffer (0.1 M) was used at a pH range of 6.5–10.0. For the optimum buffer concentration searching, phosphate buffer (pH 8.8) was used at a range of 0–200 mM.

Protein contents in the crude ALDH extraction: Lowry method [21,22] was adapted to determine protein content with Folin and Ciocalteu’s phenol reagent and bovine serum albumin was used as the standard.

#### 2.4.3. Activity of ADH in Crude Protein Extractions and Inhibition by 4-Methyl Pyrazole

To confirm the purity of ALDH, and exclude the potential influence from other enzymes, especially ADH, the ADH activity in the crude protein extraction listed in Section 2.4.1 and Section 2.4.2 was determined by monitoring the decline in NADH at 340 nm at 35 °C by a spectrophotometer in a mixture comprised of 1660 µL of phosphate buffer (0.2 M, pH 8.8), 100 µL of butanol (50 mM), 100 µL of crude protein extraction, and 40 µL of NAD^+^ (50 mM) with or without 100 µL of 4-methyl pyrazole (100 mM), where the total volume was 2 mL (balanced with water to make the volume). An extinction coefficient of 6.2 mM^−1^ cm^−1^ was used for the activity calculation [11,19].

### 2.5. Statistical Analysis

Data were present as the means and standard deviations of three replicates. Statistical analysis was performed with JMP software (version 11.2.2; SAS Institute, Cary, NC, USA). Differences were tested using Tukey’s honestly significant difference (HSD) with the significance level at 0.05.

## 3. Results

### 3.1. Feeding Alcohols to Banana Pulps to Produce Esters

**Figure 3:**
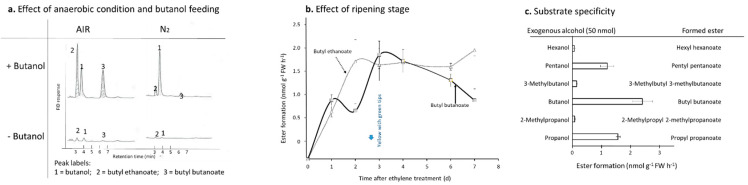
Alcohol feeding experiments and production of corresponding esters in banana pulps. Exogenous alcohol (50 µmol) on filter paper was fed into 10 g of banana pulp tissues. (**a**) Effect of anaerobic conditions and butanol feeding to pulps at the “yellow with green tips” stage; (**b**) effect of ripening stage; (**c**) substrate specificity of C3–C6 branched and straight chain alcohols at the “yellow with green tips” stage. Vertical/horizontal line at each marker/column shows average ± SD (n = 3).

When butanol vapor was fed to banana pulp slices (at the “yellow with green tips” stage), butyl ethanoate as well as butyl butanoate were accumulated (Figure 3a). Under anaerobic conditions, those esters were barely accumulated (Figure 3a). Green banana (immediately after ripening was triggered by ethylene) pulp was not able to biosynthesize such esters, but obtained the capacity within one day (Figure 3b). Butyl butanoate production increased continually until the “yellow with green tips” stage (day 3 after ethylene treatment), then gradually declined toward senescence (Figure 3b). Similarly, feeding butanol to fruit pulp of ripe muskmelon, pineapple, pawpaw, strawberry, European pear, and apple, also produced butyl butanoate (data not shown). Figure 3a,b results were confirmed when incubating a banana finger in a 1 L jar with butanol vapor, with a much lower yield (about 20%) of butyl butanoate in comparison to that of pulp slices (data not shown). When feeding other C3–C6 branched or straight chain alcohols to banana pulp instead of butanol, propyl propionate and pentyl pentanoate were produced in comparable amounts to butyl butanoate, but productions of hexyl hexanoate, 3-methylbutyl 3-methylbutanoate, and 2-methylpropyl 2-methylpropanoate were much lower (Figure 3c).

### 3.2. Feeding Aldehydes to Banana Pulps to Produce the Corresponding Acids

**Figure 4:**
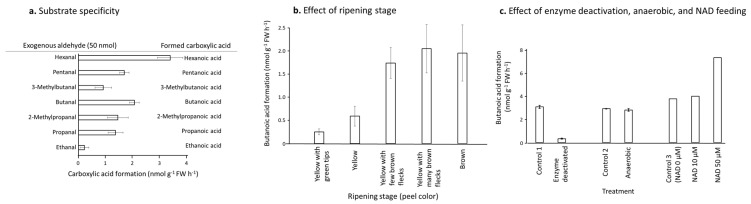
Aldehyde feeding experiments and production of corresponding carbolic acids in banana pulps. Exogenous aldehyde (50 µmol) on filter paper was fed into 10 g of banana pulp tissues. (**a**) Substrate specificity of C2–C6 branched and straight chain aldehydes at the “yellow with green tips” stage (banana pulp slices); (**b**) Effect of ripening stage (pulp homogenate); (**c**) Effects of enzyme deactivation by heat, anaerobic incubation, and feeding of NAD+ at different levels (pulp homogenate). Vertical/horizontal line at each column shows average ± SD (n = 3).

When feeding C2–C6 branched and straight chain aldehydes to the banana pulp slices (at the “yellow with green tips” stage), the acids with the highest production were hexanoic acid, followed by butanoic acid, pentanoic acid, 2-methylpropanoic acid, propanoic acid, and 3-methylbutanoic acid. The acid that was produced in the lowest quantities was ethanoic acid (Figure 4a).

When feeding butanal to banana pulp homogenate samples from fruit from different ripening stages, butanoic acid produced more in over ripe fruit—“yellow with brown flecks” and “brown” stages—and less in the “yellow” stage fruit. Butanoic acid production in fruit at the “yellow with green tips” stage was even lower (Figure 4b).

To confirm the ALDH activity in pulp homogenates, fruit at the “yellow with brown flecks” stage was combined with butanal feeding experiments. Little butanoic acid was detected when the banana pulp homogenate was heated with a 600 W microwave oven for 1 min (Figure 4c). However, anaerobic conditions did not affect the butanoic acid production (Figure 4c). When adding 50 µM NAD^+^ to the reaction system, butanoic acid production increased by two-fold in comparison to the addition of 10 µM NAD^+^, which was no different from non-NAD^+^ addition control (Figure 4c).

### 3.3. Extraction and Functional Characteristics of ALDH

**Figure 5:**
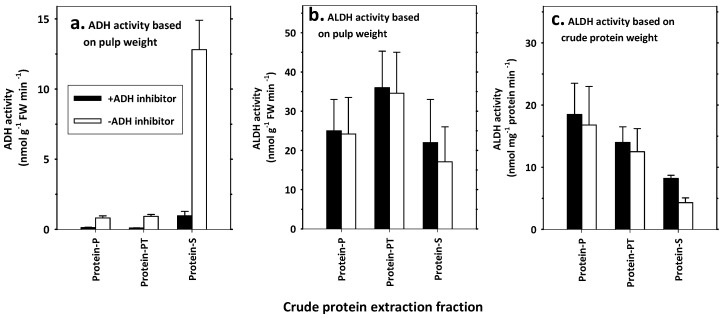
ADH and ALDH activity in crude proteins extracted from different fractions and effect of Triton X-100 (protein extraction enhancer) and 4-methyl pyrazole (ADH inhibitor). Proteins were extracted by ammonium sulfate precipitation from Tris buffer extraction of banana pulp tissues at the “yellow with brown flecks” stage. The activity was presented based on fresh sample mass (**a**,**b**) or protein mass (**c**). Protein-S: extraction from supernatant merged from 2 × Tris buffer extractions; protein-P: extraction from pellets was washed 2 × Tris buffer and solubilized by sonication; protein-PT: extraction from pellets after 2 × Tris buffer washing and solubilized by sonication with addition of Triton X-100. Vertical line at each column shows average ± SD (n = 3).

As shown in materials and methods, crude proteins were extracted in three ways: from supernatant (protein-S), or from pellets by sonication-assisted extraction with or without Triton X-100 (protein-PT and protein-P, respectively). The protein-S possessed more than 95% of ADH activity, while both protein-P and protein-PT had very little ADH activity (Figure 5a). On the other hand, ALDH activity was evidenced in all protein extractions (Figure 5b,c).

When based on weight of pulp tissue, calculated ALDH activity distribution for protein-P was slightly more than for protein-S; however, the distribution at pellet extraction was increased when Triton X-100 was used (protein-PT) (Figure 5b). When 4-methyl pyrazole was added into the reaction mixture, ALDH activity distribution at protein-PT was about 60%, and at protein-S, it was about 40% (Figure 5b). However, when calculation was based on protein weight, the highest ALDH activity was found in protein-P, followed by protein-PT and then protein-S. This was partially because Triton X-100 caused more extraction in non-ALDH proteins (protein-PT), and protein-S contained more non-ALDH proteins (Figure 5b,c). ALDH-PT contained more yellow pigments as contaminants. Protein-S had the lowest ALDH activity at protein basis (Figure 5). Thus, protein-P was chosen as crude extract of ALDH in the following experiments.

**Figure 6:**
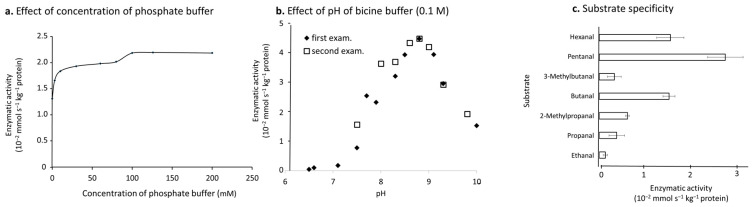
Activity of crude ALDH. (**a**) Optimum concentration of phosphate buffer; (**b**) Optimum pH; and (**c**) Substrate specificity to C2–C6 branched and straight chain aldehydes. Horizontal line at each column shows average ± SD (n = 3).

ALDH activity was very low when the phosphate buffer concentration was less than 20 mM (Figure 6a). The activity increased slightly until the buffer concentration reached saturation at 100 mM (Figure 6a). Optimum pH of the ALDH was 8.8 as shown in Figure 6b, and at pH 8.5–9.2, ALDH activities were kept > 80% capacity (Figure 6b). Km values of butanal and NAD^+^ of the ALDH were about 250 μM and 25 μM, respectively, from the Lineweaver–Burk plot (data not shown). When replacing NAD^+^ by NADP^+^, the ALDH activity reduced to about one third in comparison to NAD^+^ (data not shown).

Substrate specificity of the ALDH was shown in Figure 6c. Lower molecular weight straight chain aldehydes, except ethanal, had high affinity to ALDH, while poor affinity was detected for branched chain aldehydes (Figure 6c).

### 3.4. Feeding 2-Keto-4-Methylpentanoic Acid to Banana Pulps to Produce Branched Alcohol, Aldehyde and Ester

**Figure 7:**
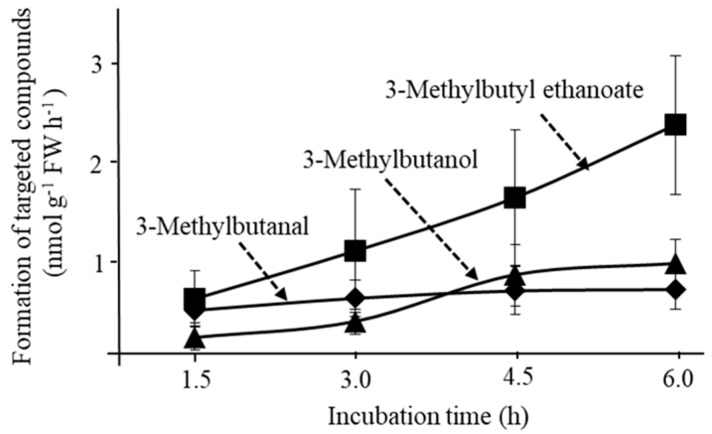
Formation of 3-methylbutanol, 3-methylbutanal and 3-methylbutyl ethanoate from exogenous 2-keto-4-methylpentanoic acid (50 nmol) after incubation with pulp slices (10 g) of bananas at the “yellow with green tips” stage. Vertical line at each marker shows average ± standard deviation (n = 3).

To test the production of branched chain alcohol, aldehyde and ester from keto acid, 2-keto-4-methylpentanoic acid was fed to banana pulps. As expected, 3-methylbutanol and 3-methylbutal were detected in the headspace (Figure 2a). However, there was no 3-methylbutyl 3-methybutanoate in the headspace, although 3-methylbutyl ethanoate was detected (Figure 7).

## 4. Discussion

Under anaerobic conditions, exogenous butanol fed to banana pulp was not effectively converted to butyl butanoate (Figure 3a), indicating that oxygen was required in the pathway from alcohols to esters (Figure 1). Due to ADH, ACS, and AAT all not directly requiring oxygen, aldehyde oxide (ALO) was suspected as the enzyme which catalyzes butanal to butanoic acid, although only several specific substrates were catalyzed by ALO in plants, some of which are precursors of important plant hormones (indole-3-acetate (IAA) and abscisic acid (ABA)) [23] (Figure 1). However, the butanal feeding experiment showed that oxygen was not directly required; under anaerobic conditions, banana pulp converted exogenous butanal to butanoic acid, meanwhile, NAD^+^ was the cofactor (Figure 4c), indicating that ALDH, not ALO, is the enzyme. Another side-by-side experiment in Figure 4c showed that enzyme-deactivated pulp tissue by microwave heating lost the ability to convert butanal to butanoic acid. Nevertheless, there is not a clear answer why under anaerobic conditions, feeding with butanol did not produce butyl butanoate (Figure 3a). It seems that a deficit of NAD^+^ may correlate to the cascade conversions from alcohols to carboxylate, and dehydrogenation of NADH to NAD^+^ was blocked under anaerobic conditions (Figure 1).

In vitro experiments showed that the optimum phosphate buffer concentration to ALDH was >100 mM (Figure 6a). This might be due to phosphate ion accelerating the activity of ALDH, which was observed in germinating peanut cotyledon [24]. NAD^+^ was a better coenzyme to ALDH and the efficiency of NADP^+^ was only one third in comparison to NAD^+^ (data not shown), although NAD^+^ and NADP^+^ perform similar redox functions within the cell. The latter is also more confined to biosynthetic pathways and redox protective roles in general [25].

During ripening, ALDH activity in the homogenate increased continually until the “yellow with many brown flecks” stage, and then it remained unchanged or slightly decreased in the butanal feeding experiment (Figure 4b). However, when butanol was fed into banana slices at different ripening stages, the peak of butyl butanoate production appeared the “yellow with green tips” stage (Figure 3b), indicating that experiments using pulp slices and homogenate may lead to different results in enzyme activities: ALDH could have high activity at younger ripening stage, i.e., at the “yellow with green tips” stage (Figure 3b), but at that stage, polyphenol and tannin contents were much higher than at the “yellow with many brown flecks” stage, which were mixed with enzymes during homogenization, thus inhibiting the enzyme activity of ALDH (Figure 4b) [26].

In the substrate specificity experiments feeding C2–C6 branched or straight chain aldehydes, the ones with the highest affinity to ALDH were even and straight chain C6 and C4 aldehydes, and that with the lowest was C2 ethanal (Figure 4a). This means that ethanal formed during fermentation or senescence cannot leak to ethanoic acid, which usually is a key function in detoxification of exogenously and endogenously generated aldehydes in mammals [27]. Branched chain aldehydes were generally lower in affinity to ALDH in comparison to the straight chain aldehydes with the same carbon number (Figure 4a). The trends are similar to the affinity of the acyl-CoA/carboxylate to AAT [14]: the even and straight chain substrates had high affinity, but branched chain substrates had low affinity (Figure 4a). A conflicting observation was that even though the affinity of ALDH for hexanal was high (Figure 4a), very low conversion occurred from exogenous hexanol to hexyl hexanoate (Figure 3c). One of the possible reasons is due to slow partition of hexyl hexanoate in headspace, it did not build up enough vapor when direct headspace sampling was used, even though hexyl hexanoate was abundant in the pulps or solutions. For example, hexyl hexanoate was one of the most abundant esters in Gala apples when Tenax GC trap or solid-phase microextraction (SPME) trap sampling methods were used [28]. However, when direct headspace sampling method was used, hexyl hexanoate was not detectable [29].

TCA cycle and β-oxidation of fatty acids in fruit pulps continually provide ethanoyl-CoA (acetyl-CoA)/ethanoate to the background in the pulp. One of the consequences is that when feeding ethanol, numerous ethyl ethanoates are produced, and it is difficult to differentiate whether the ethanoyl-CoA/ethanoate are from the fed ethanol or the background substrates. Thus, in Figure 3c, ethanol feeding was not shown. Thus, in bananas, there are rich sources of ethanoate/ethanoyl-CoA via TCA and β-oxidation, and ALDH is not the major way to produce ethanoate (Figure 4a).

It is well known that butanoate esters are the second most produced esters after ethanoate esters in banana fruits [30], and the high affinity of butanal to ALDH ensured sufficient butanoate (Figure 4a). However, ester profiles changed with fruit senescence [30,31], controlled/modified atmosphere [32,33,34], or other treatments which may extend shelf life with sacrifice of flavor quality [29]. Due to ester production being more dependent on alcohol and carboxylic acid substrate availability than substrate specificity of AAT [14,15,35], ALDH plays a key role in the ester profile and flavor quality of fresh fruit.

Feeding of 2-keto-4-methylpentanoic acid to banana pulps produced 3-methylbutanol, and 3-methylbutyl ethanoate, but no 3-methylbutanoate and the esters (Figure 7). The results confirmed that the affinity of ALDH for branched chain aldehydes is low. There are reports that some fruits such as apples and melons produce a relatively high amount of esters consisting of branched chain carboxylic acid [36,37]. It is worth continuing research on ALDH in branched chain carboxylic acid rich fruits.

The most critical question for this feeding model research was how to obtain high purity ALDH and minimize the effect of other enzymes, especially ADH, which is also determined by monitoring the change in NADH concentration. Furthermore, ADH facilitates the interconversion between alcohols and aldehydes with the redox between NAD^+^ and NADH. In this study, two actions were taken to eliminate the potential effect of ADH. The first was to remove proteins that had high ADH activity but much less ALDH activity. We removed elutable proteins (protein-S) which contained 95% of ADH, but only about 40% of ALDH activity, and obtained proteins which had higher binding force to pellets—they were extracted under sonicator-assistance (Figure 5). In such pellet protein, there was little ADH activity (Figure 5). The second action involved using 4-methyl pyrazole, an ADH inhibitor, to block any potential ADH activity in the reaction mixture for ALDH activity determination (Figure 5).

## 5. Conclusions

ALDH, which converts aldehydes to carboxylic acids, was found in banana pulps and may play a key role in the conversion between alcohols, aldehydes, carboxylic acids and esters, and the formation of fruit aromas. Crude ALDH tests showed that the enzyme required NAD+ as a cofactor, and the optimum pH was 8.8. Lower molecular weight straight chain aldehydes, except ethanal, had high affinity to ALDH, while poor affinity was detected to branched chain aldehydes. Further research is needed to confirm whether ALDH is an enzyme in the routine pathway for volatile production associated with fruit ripening or just a consequence of aldehyde scavenging.

## Data Availability

Data is contained within the article.
